# Application of functional near-infrared spectroscopy (fNIRS) in tinnitus research: contemporary insights and perspectives

**DOI:** 10.3389/fpsyg.2024.1334660

**Published:** 2024-02-02

**Authors:** Hantong Hu, Xiaoqi Lin, Ziyu Ye, Lianqiang Fang, Hong Gao, Quanai Zhang

**Affiliations:** ^1^Department of Acupuncture and Moxibustion, The Third Affiliated Hospital of Zhejiang Chinese Medical University, Hangzhou, China; ^2^Department of Neurobiology and Acupuncture Research, Key Laboratory of Acupuncture and Neurology of Zhejiang Province, Zhejiang Chinese Medical University, Hangzhou, China; ^3^The Third Clinical Medical College, Zhejiang Chinese Medical University, Hangzhou, China; ^4^Clinical Medical College of Acupuncture, Moxibustion and Rehabilitation, Guangzhou University of Chinese Medicine, Guangzhou, China

**Keywords:** tinnitus, phantom sound, fNIRS, neuroimaging, brain plasticity

## Abstract

Tinnitus, characterized by phantom sound perception, is a highly disruptive condition lacking clearly effective treatments. Its complex neural mechanisms are not fully elucidated. Functional near-infrared spectroscopy (fNIRS) is a promising neuroimaging tool well-suited for assessing tinnitus due to its quietness, portability, and ability to directly measure cortical hemodynamic responses. This study timely summarizes the recent applications of fNIRS in investigating tinnitus pathology, correlating neuroimaging biomarkers with symptom severity, and evaluating treatment efficacy. Further studies with larger samples are warranted to reproduce existing findings. Thus, fNIRS appears to be a promising tool in tinnitus research. Addressing technical limitations, optimizing control groups, advancing data analysis, integrating standardized, and individualized experimental protocols can facilitate the extended and robust utilization of fNIRS in tinnitus research.

## 1 Introduction

Tinnitus is defined as the perception of sound when no external noise is present (McCormack et al., 2016). Epidemiological surveys indicate that 10–15% of the general population suffer from troublesome tinnitus, with prevalence increasing with age and expected to rise in the future due to growing noise exposure (Kim et al., 2015; McCormack et al., 2016). Tinnitus can significantly disrupt sleep and concentration, cause emotional distress and psychiatric comorbidities, and severely impair quality of life (Bhatt et al., 2017). Currently no universally effective treatments are available (Cima et al., 2019).

The neural mechanisms underlying tinnitus have been investigated for decades yet remain poorly defined. Contemporary models postulate that various insults to the peripheral auditory system trigger a series of maladaptive plastic changes in the central nervous system, causing hyperactivity and hyperconnectivity that eventually result in aberrant spontaneous firing interpreted as phantom sounds (Noreña and Eggermont, 2003). Animal studies support this “central neural gain” (i.e., increased spontaneous neural firing rates and neural synchrony) model within auditory cortex (Auerbach et al., 2014; Takacs et al., 2017; Hayes et al., 2021). In humans, altered spontaneous neural rhythms (Weisz et al., 2005; Eggermont and Tass, 2015) and functional connectivity (Job et al., 2020) have been detected in individuals with chronic tinnitus using functional magnetic resonance imaging (fMRI). Still, the specific brain networks and dynamics underlying the pathogenesis of tinnitus remain inconclusive.

Non-invasive neuroimaging techniques are crucial for advancing the understanding of tinnitus pathology in humans. The current techniques used in tinnitus neuroimaging research each have merits and limitations. Electroencephalography (EEG) directly measures electrical potentials with high temporal resolution, but has low spatial specificity in localizing activation sources (Beniczky and Schomer, 2020). Magnetoencephalography (MEG) is a technique that measures magnetic fields produced by electrical currents in the brain (Kim and Davis, 2021), achieving enhanced source localization yet constrained by exorbitant costs. fMRI measures blood oxygen level dependent (BOLD) signals but is susceptible to interference from scanner noise (Glover, 2011; Schramm et al., 2023). These constraints underscore the need for novel neuroimaging tools tailored for tinnitus assessment.

### 1.1 Functional near-infrared spectroscopy (fNIRS) as an emerging tool for tinnitus

Functional near-infrared spectroscopy (fNIRS) is an emerging neuroimaging modality gaining increasing attention in tinnitus research. It uses near-infrared light to measure cortical hemodynamic responses in brain regions of interest (ROI), considered an indirect marker of local neural activation (Pinti et al., 2020). The physical basis is that near-infrared light penetrates biological tissues, while hemoglobin (Hb) chromophores in red blood cells absorb certain near-infrared wavelengths depending on their oxygenation state. By emitting near-infrared light into the scalp and detecting its absorption at specific wavelengths, fNIRS systems can quantify changes in oxygenated hemoglobin (HbO2) and deoxygenated hemoglobin (dHb) levels, which arise from neurovascular coupling of local cortical activation (Kim et al., 2017).

fNIRS offers several advantages tailored for assessing tinnitus (Basura et al., 2018). The portability of fNIRS systems allow for more controlled experimental setups. The quiet optical recordings avoid interference with auditory processes. It is relatively low-cost and does not involve radiation exposure. The temporal resolution, while lower than EEG/MEG, captures hemodynamic changes on a timescale suited for many auditory and cognitive paradigms. These merits make fNIRS suited for tinnitus research.

In recent decades, fNIRS has been widely applied to investigate the central mechanisms underlying tinnitus and changes in cortical functions after treatment. Thus, our study summarizes recent research advancements in using fNIRS for tinnitus studies and discuss prospects to address current research gaps.

## 2 Application of fNIRS in tinnitus research

We conducted a literature search in PubMed, Web of Science, and ScienceDirect in October 2023. The search strategy was based on the combination of the following keywords: “Functional Near-Infrared Spectroscopy,” “fNIRS,” “NIRS” “neuroimaging,” “tinnitus,” and “auditory.” For detailed search strategies, literature eligibility criteria, and the PRISMA flow diagram for literature screening were shown in Supplementary materials. On this basis, to the best of our knowledge, research on the application of fNIRS in tinnitus research is still limited, with only a few studies published. Table 1 summarizes the key characteristics of the analyzed studies.

**Table 1:** The key characteristics of the analyzed fNIRS studies in tinnitus research.

Study	Study population	Control	Main findings
Schecklmann et al. (2014)	Chronic tinnitus adults (tinnitus duration: 68.9 ± 61.4 months), (*n* = 12) who received rTMS (10 sessions)	1. Chronic tinnitus adults (*n* = 11; tinnitus duration: 96.8 ± 120.4 months) who received sham rTMS (10 sessions); 2. Healthy control group (non-tinnitus participants, *n* = 12)	1. Block-design and event-design paradigms in fNIRS resulted in different patterns of activation in auditory and temporoparietal cortices in tinnitus patients. Findings indicate the sensitivity of fNIRS for detecting rTMS induced changes in brain activity and the potential of fNIRS for the investigation of tinnitus pathophysiology.
Issa et al. (2016)	Chronic tinnitus adults with normal/near-normal hearing (*n* = 10)	Non-tinnitus controls with normal/near-normal hearing (*n* = 7)	Following sound stimulation, nontinnitus controls demonstrated deactivation in both auditory and nonauditory regions during inter-stimulus silent periods, while tinnitus participants demonstrated maintenance of activation
San Juan et al. (2017)	Chronic bilateral tinnitus adults with normal/near-normal hearing (*n* = 10)	Non-tinnitus adults with normal/near-normal hearing (*n* = 8)	Following sound stimulation, RSFC of both auditory cortex and non-auditory cortices increased in tinnitus participants but decreased in non-tinnitus controls.
Shoushtarian et al. (2020)	Chronic tinnitus individuals with normal/near-normal hearing (*n* = 25; tinnitus duration: 11.5 ± 8.8 years)	Non-tinnitus controls with normal/near-normal hearing (*n* = 21)	Findings supported the feasibility of using the combination of fNIRS and machine leaning to identify and predict the severity of tinnitus. It also suggests fNIRS-derived neuroimaging markers can objectively reflect tinnitus perceptual features and potentially serves as an objective measure of tinnitus.
Verma et al. (2019)	Bilateral tinnitus patient (*n* = 1, duration: 2 years) with profound sensory neural hearing loss (>100 dB) in the right ear and mild conductive hearing loss in the left ear; and received tDCS (20 session)	Not applicable	1. There was significant improvement in tinnitus symptoms and functional cortical activity after 20 sessions of tDCS. 2. fNIRS can be a tool to evaluate the theraputic effect of tDCS.
Yu et al. (2023)	Bilateral tinnitus participants with normal/near-normal hearing (*n* = 18, tinnitus duration: 20.44 months) who received acupuncture (10 sessions)	Not applicable	Acupuncture increased the concentration of oxygenated hemoglobin in the temporal lobe of tinnitus patients and affected the activation of the auditory cortex.
Hu et al. (2023)	Tinnitus patients randomly assigned to the experimental group (*n* = 30); and received acupuncture (12 sessions)	Tinnitus patients randomly assigned to the waiting-list control group (*n* = 30); and receive no intervention	Not applicable (note: this RCT is still ongoing with no published results and findings)
Sun et al. (2020)	Tinnitus patients (*n* = 13, tinnitus duration: 16.31 months; tinnitus frequency: between 4,000 and 8,000 Hz), and recived three masking sounds as acoustic stimuli (one session)	Non-tinnitus controls (*n* = 20)	fNIRS showed three cortical activation patterns induced by three different masking noises, and correlations between residual inhibition effects and change of HbO amplitude were found. fNIRS can be adopted as an objective measure for assessing the efficacy of acoustic therapy.
Huang et al. (2021)	Ttinnitus patients with normal hearing or mild hearing loss (*n* = 29; tinnitus duration: 10.90 ± 9.34 months; tinnitus frequency: between 4,000 and 8,000 Hz); treated with notched sound (30 sessions)	Not applicable	Cerebral cortex reorganization occurs in tinnitus patients after notched sound treatment, and notched sound decreases the connectivity between the auditory cortex and specific brain regions.

fNIRS, functional near-infrared spectroscopy; rTMS, repetitive transcranial magnetic stimulation; RSFC, resting state functional connectivity; tDCS, transcranial direct current stimulation.

### 2.1 Investigate central mechanisms of tinnitus using fNIRS

Schecklmann et al. (2014) first used fNIRS in tinnitus research to investigate central mechanisms of tinnitus, comparing non-tinnitus controls to chronic tinnitus patients undergoing rTMS or placebo rTMS. Tinnitus patients showed elevated right auditory cortex excitation with block stimuli and diminished frontal excitation with event-related stimuli at baseline versus controls. Oxygenation patterns flipped post-rTMS versus placebo. These results demonstrate the sensitivity of fNIRS in detecting cerebral changes in tinnitus patients. To note, this study establishes a foundation for employing fNIRS to investigate central mechanisms of tinnitus. It also highlights the feasibility and potential of fNIRS in tinnitus research for the first time.

Later, a study used fNIRS to Issa et al. (2016) evaluate hemodynamic responses in auditory cortex and adjacent extra-auditory cortices in tinnitus patients with normal hearing and healthy controls (i.e., non-tinnitus participants with normal hearing). Stimuli included 750 Hz, 8,000 Hz tones and broadband noise, with silence during intervals. Results showed hemodynamic response in auditory cortex and extra-auditory cortices were deactivated during periods of silence in non-tinnitus participants, while tinnitus patients maintained increased hemodynamic responses. Increased hemodynamic responses in extra-auditory cortices also aligns with previously observed adaptive changes (e.g., altered connectivity and network activity, neuroplastic changes) in extra-auditory regions in tinnitus patients.

Another study (San Juan et al., 2017) utilized fNIRS to examine alterations in resting state functional connectivity (RSFC) between auditory and extra-auditory cerebral regions in tinnitus patients and non-tinnitus controls. RSFC was assessed using fNIRS during 60 s of quiet rest periods before and after listening to alternating pure tones of 750 Hz and 8,000 Hz, as well as broadband noise. Prior to auditory exposure, the auditory cortex showed reduced connectivity to temporal and fronto-temporal areas in tinnitus patients compared to non-tinnitus controls. After auditory stimulus, the connectivity patterns in tinnitus patients diverged notably from non-tinnitus controls. There was observed augmentation in connectivity within both auditory and extra-auditory regions for the tinnitus patients, whereas non-tinnitus participants exhibited diminished connectivity. Specifically, in tinnitus sufferers, the intensity of RSFC heightened between the auditory cortex and regions including the fronto-temporal, fronto-parietal, occipito-temporal, temporal, and occipital cortices. Cumulatively, their findings propose that both auditory and extra-auditory cerebral regions undergo alterations in tinnitus. Moreover, fNIRS could act as a promising tool for providing objective measurement for tinnitus.

In short, the above pioneering fNIRS studies established feasibility of the technique in revealing potential tinnitus neural correlates, including elevated spontaneous firing rates and abnormal cross-network functional connectivity. The current findings should be reproduced in larger patient cohorts. Additionally, investigating possible subgroups based on tinnitus features could better elucidate the heterogeneous mechanisms of chronic tinnitus.

### 2.2 Assessing the severity of tinnitus using fNIRS

To note, the objective assessment of tinnitus symptom severity and the accurate subtyping of patients are critical yet unaddressed challenges in the field. Nonetheless, with the progressive application of fNIRS and machine learning in tinnitus research, it is anticipated that these challenges can be partially addressed.

Through machine learning applied to functional connectivity between cortical regions measured by fNIRS, a study (Shoushtarian et al., 2020) attempted to correlate the strength of functional connectivity with the tinnitus severity. In detail, they examined 25 chronic tinnitus individuals and 21 non-tinnitus controls using fNIRS. Results showed higher temporal-temporo-parietal connectivity at rest in tinnitus patients versus controls. Connectivity increased markedly with subjective loudness ratings within the tinnitus group. Tinnitus patients also demonstrated reduced region-specific visual and auditory responses. Furthermore, multiple machine learning algorithms were utilized to learn features like auditory/visual response amplitudes and fronto-temporo-parietal connectivity, attempting to categorize tinnitus severity. A naive Bayes classifier achieved 78.3% accuracy in distinguishing tinnitus from controls. Using neural networks, tinnitus severity classification into mild/moderate/severe categories had 87.32% accuracy.

In brief, this study supported the feasibility of using the combination of fNIRS and machine leaning to identify and predict the severity of tinnitus. It also suggests fNIRS-derived neuroimaging markers can objectively reflect tinnitus perceptual features and potentially serves as an objective measure of tinnitus. However, this study was limited by the small sample size. Therefore, large-scale studies are warranted to verify the reliability of fNIRS-derived neuroimaging markers for assessing tinnitus severity.

### 2.3 Evaluating the treatment effect and mechanisms underlying specific therapies using fNIRS

fNIRS is emerging as a powerful neuroimaging technique to examine the therapeutic effects and mechanisms of various tinnitus treatments. By monitoring real-time changes in HbO2 levels and functional connectivity patterns in relevant cortical regions associated with treatment response, these studies shed light on the value of fNIRS as an instrument for objective assessment of tinnitus treatments, as well as investigate the neural mechanisms underlying these therapies for treating tinnitus.

#### 2.3.1 Transcranial direct current stimulation (tDCS)

A previous case study (Verma et al., 2019) utilized fNIRS to evaluate the therapeutic effect of tDCS and confirmed significant improvement in functional cortical activity following 20 sessions of tDCS in a patient with bilateral tinnitus for 2 years. This improvement positively correlated with alleviated tinnitus symptoms. However, as a single case report, the findings have limited generalizability and should be interpreted cautiously, requiring more robust research for validation.

#### 2.3.2 Acupuncture

One study (Yu et al., 2023) recruited 18 tinnitus subjects and outcomes measured by fNIRS included the HbO2 concentration, the dHb concentration, and the activation of multiple channels during sound-evoked activity. Results revealed that acupuncture increased the HbO2 in the temporal lobe. Moreover, the superior temporal gyrus showed significant activation after acupuncture, revealing that acupuncture can modulated activation of the auditory cortex in tinnitus patients. Their findings provide insight into the central mechanisms underlying the therapeutic effects of acupuncture for tinnitus and may facilitate objective evaluation of acupuncture efficacy.

The other study (Hu et al., 2023) reported a study protocol of a randomized controlled trial (RCT) that used fNIRS to evaluate the efficacy of acupuncture for chronic tinnitus. Sixty patients would be randomized into the acupuncture group or a waiting-list control group. Changes in brain oxygenation and functional connectivity in brain regions associated with tinnitus were examined using fNIRS. Specifically, the brain regions of interest examined with fNIRS included the primary auditory cortex, the ventromedial prefrontal cortex, and the dorsolateral prefrontal cortex. However, this RCT is still ongoing with no published data.

#### 2.3.3 Sound therapy

One study (Sun et al., 2020) used fNIRS to investigate the cortical effects of sound therapy on tinnitus patients using three spectrally distinct masking sounds (i.e., white noise, narrow-band noise, notched sound). The study included 20 non-tinnitus controls and 13 tinnitus patients. Results showed that the narrow-band noise notably decreased HbO2 in the Brodmann area 21 (BA21) region, whereas the white noise led to an increase in HbO2 levels, and notched sound demonstrated minimal impact. Although many studies have tested the efficacy of different masking sounds for tinnitus, there is no consensus on the optimal sound type. By observing distinct cortical activation patterns using fNIRS associated with residual inhibition, this study demonstrates the feasibility and potential of fNIRS as an objective measure for assessing the efficacy of sound therapy.

The other study (Huang et al., 2021) used fNIRS to assessed changes in resting cortical function before and after 1-month notched sound treatment in 21 tinnitus patients. Results indicate that notched sound treatment induced stable cortical reorganization. Besides influencing the auditory cortex, notched sound markedly reduced the connectivity of Brodmann area 46 (BA46). Previous studies have linked BA46 to emotion processing, identifying it as a region involved in the inhibition of negative affect (Aron et al., 2014). This provides evidence that notched sound improves tinnitus by regulating both auditory and emotional cortex.

## 3 Discussion

This study timely summarizes the emerging application of NIRS in tinnitus research over recent years. The current findings suggest fNIRS holds promise to advance understanding of the complex mechanisms underlying tinnitus, assess symptom severity, and provide objective measures of treatment efficacy for various therapies. However, further studies are necessary to consolidate these findings and expand their clinical applicability.

### 3.1 Challenges and future directions

While great potential has been demonstrated, the use of fNIRS in tinnitus research is still in its nascent stages. Realizing the full potential of fNIRS in tinnitus research requires overcoming its current technical limitations and various challenges.

#### 3.1.1 Addressing technical limitations

The widespread application of fNIRS faces several key obstacles related to its technical limitations: suboptimal spatial and temporal resolution, limited penetration depth, and relatively low reproducibility. To realize the full potential of fNIRS in tinnitus research, overcoming or compensating these technical limitations is crucial.

First, better spatial resolution of fNIRS plays an important role in tinnitus research for improving anatomical specificity, thereby identifying specific brain regions associated with tinnitus pathology. Enhancing the spatial resolution of fNIRS can be achieved through its co-registration with other neuroimaging modalities, such as fMRI and EEG. Such integration combines the strengths of each modality: the superior spatial resolution of fMRI, the temporal resolution of EEG, and the real-time hemodynamic response monitored by fNIRS, so this multi-modal approach becomes a major trend for brain monitoring (Phillips et al., 2023). Although multi-modal brain monitoring remains lacking in tinnitus research, valuable insights and methodologies can be derived from various subfields of neuroscience. For instance, a study (Amyot et al., 2020) demonstrated that the combined use of fNIRS and fMRI enhances better spatial and temporal resolution in patients with traumatic cerebral vascular injury. Another study (Blanco et al., 2023) highlighted the complementarity between fNIRS and EGG for monitoring different brain states. In addition, multichannel optode caps have emerged as a method to improve spatial resolution. Yet, these caps can be uncomfortable, affecting data quality and the ease of use. Thus, future research should explore new materials and configurations that can offer high-density sensor placement without compromising on the wearability and convenience of the fNIRS device. Notably, high-density arrays of near-infrared sources and detectors have recently demonstrated substantial enhancements in spatial resolution (Frijia et al., 2021).

Another limitation of fNIRS is its inability to access deeper cortical regions, given the restriction of only approximately 3 cm penetration depth of current methodologies (Ayaz et al., 2013). However, brain changes associated with tinnitus pathogenesis involve deeper regions of the cerebral cortex (e.g., the cingulate cortex, amygdala, and insula) that are beyond the measurable range of conventional fNIRS configurations. To address this constraint, adapted fNIRS techniques deserve further investigation. For example, a study (Zhai et al., 2021) reported an adapted fNIRS approach that can circumvent the obstructive cranial bone by strategically placing specialized probes closer to the temporal lobe, which offers a promising direction for accessing these deeper regions.

An additional technical aspect of fNIRS research in tinnitus involves enhancing signal quality. The arrangement of emitters and detectors in fNIRS caps is pivotal for signal quality. Recent advancements have focused on optimizing emitter-detector arrangements to improve signal quality. For example, high-density fNIRS arrays with closer emitter-detector proximity can increase spatial resolution and reduce scalp interference. Furthermore, incorporating short-distance channels as a reference distinguishes cerebral from extracerebral signals, thereby improving measurement accuracy (Sanchez-Alonso et al., 2023).

#### 3.1.2 Optimizing control groups

Selecting an appropriate control group is crucial in RCTs or cross-sectional studies. Since tinnitus often co-occurs with conditions like hearing loss, hyperacusis, or depression, determining the optimal control can be difficult. Earlier studies often matched controls to tinnitus groups by age and sex alone (Elgoyhen et al., 2015); and they attributed structural and functional differences to tinnitus even when the groups differed in hearing, hypersensitivity, or mood. More recent studies used controls matched for hearing loss, hyperacusis, and depression (Elgoyhen et al., 2015). To a certain extent, this allows unambiguously tying group differences to tinnitus. However, to date, the specific variables that need to be matched between tinnitus and control groups have been inconclusive. Therefore, more research is warranted to determine the optimal matching variables between control groups and tinnitus groups for yielding reliable comparative findings.

#### 3.1.3 Integrating standardized and individualized experimental protocols

Standardized protocols are vital for consistency across studies, thus enabling comparisons between different studies. However, fNIRS studies often face reproducibility challenges due to subject variability and heterogeneity in experimental protocols (Chen et al., 2020). Drawing lessons from other neuroscience fields, such as posture and gait research, where standardized fNIRS protocols have been established in a consensus guide (Menant et al., 2020), can be enlightening. The consensus provides comprehensive guidelines on paradigms of event-related or block designs, optode placement, ROI selection, and data processing and other key procedures, which can serve as a model for achieving higher reproducibility and comparability in fNIRS research.

Yet, the unique challenges in tinnitus research, characterized by its multifactorial nature in tinnitus etiology, highlight the need for individualized fNIRS protocols. This could include customizing sensor placements to cater to the specific neural pathways affected in patients across different studies. Such individualized approaches, while challenging to standardize, could provide deeper insights into the neural underpinnings of tinnitus and further enhance the precision of clinical interventions.

In short, a hybrid strategy, integrating standardized and individualized protocols, may be a more appropriate approach in fNIRS research for tinnitus, especially during the current early stage of research in this field.

#### 3.1.4 Advancing data analysis

Prior to formally analyzing the fNIRS data, it is essential to exclude physiological noise. Physiological noise sources in fNIRS, such as scalp blood flow, blood pressure fluctuations, and heart rate, have been addressed using various noise reduction and signal processing strategies. These include prewhitening, digital filtering, adaptive filtering, and data-driven approaches like independent component analysis and principal component analysis and (Chen et al., 2020). Short separation channels have also become instrumental in mitigating scalp blood flow impact on fNIRS data (Nguyen et al., 2018).

fNIRS data analysis can benefit from artificial intelligence (AI). AI-based analysis methods such as machine learning and deep learning could decipher the vast and complex datasets, potentially unveiling subtle neural patterns associated with tinnitus. For example, a study (Shoushtarian et al., 2020) employed four kinds of machine learning algorithms to analyze fNIRS data in tinnitus patients, thereby successfully predicting the severity of tinnitus.

Notably, the strengths of machine learning and deep learning in analyzing fNIRS data can handle large datasets and complex patterns in data that are not easily discernible through traditional methods, while the limitation is that they require large datasets for training and can be prone to overfitting.

## 4 Conclusion

In summary, the advent of fNIRS in tinnitus research marks the beginning of a promising era. It has been emerging as a promising instrument to advance understanding of the heterogeneous mechanisms underlying tinnitus, provide objective measures of symptom severity, and quantify treatment efficacy. Further research and technological advancements may promote fNIRS to greater heights, rendering it increasingly suitable for tinnitus research.

## Data availability statement

The original contributions presented in the study are included in the article/Supplementary material, further inquiries can be directed to the corresponding authors.

## Author contributions

HH: Conceptualization, Writing – original draft. XL: Writing – original draft. ZY: Writing – review & editing. LF: Data curation, Writing – review & editing. HG: Writing – review & editing. QZ: Conceptualization, Supervision, Writing – review & editing.
