# Supplementary material for: Application of functional near-infrared spectroscopy (fNIRS) in tinnitus research: contemporary insights and perspectives
Source: Front Psychol. 2024 Feb 2;15:1334660. doi: 10.3389/fpsyg.2024.1334660 (PMC10870148; doi:10.3389/fpsyg.2024.1334660)
Supplement: Supplementary file 1 [file Table_1.DOCX]

**Supplementary Table1. Search strategy for each database**

| **Databases** | **Search strategy** |
| --- | --- |
| PubMed | (("Functional Near-Infrared Spectroscopy"[Title/Abstract] OR fNIRS[Title/Abstract] OR NIRS[Title/Abstract]) AND (tinnitus[Title/Abstract] OR auditory[Title/Abstract])) |
| Web of Science | TS=("Functional Near-Infrared Spectroscopy" OR fNIRS OR NIRS) AND TS=(tinnitus OR auditory) |
| ScienceDirect | ("Functional Near-Infrared Spectroscopy" OR fNIRS OR NIRS) AND (tinnitus OR auditory) |

**Supplementary Table 2. Inclusion and Exclusion Criteria for Literature**

| **Inclusion Criteria** | **Exclusion Criteria** |
| --- | --- |
| 1. Primary research articles, original studies, or preliminary reports that utilized fNIRS to investigate any aspect of tinnitus. 2. Studies conducted in humans 3. Publications published in English | 1. Review articles, conference abstracts, editorial material, letters and commentaries 2. Experimental studies that are not conducted on humans 3. Publish in non-English language |


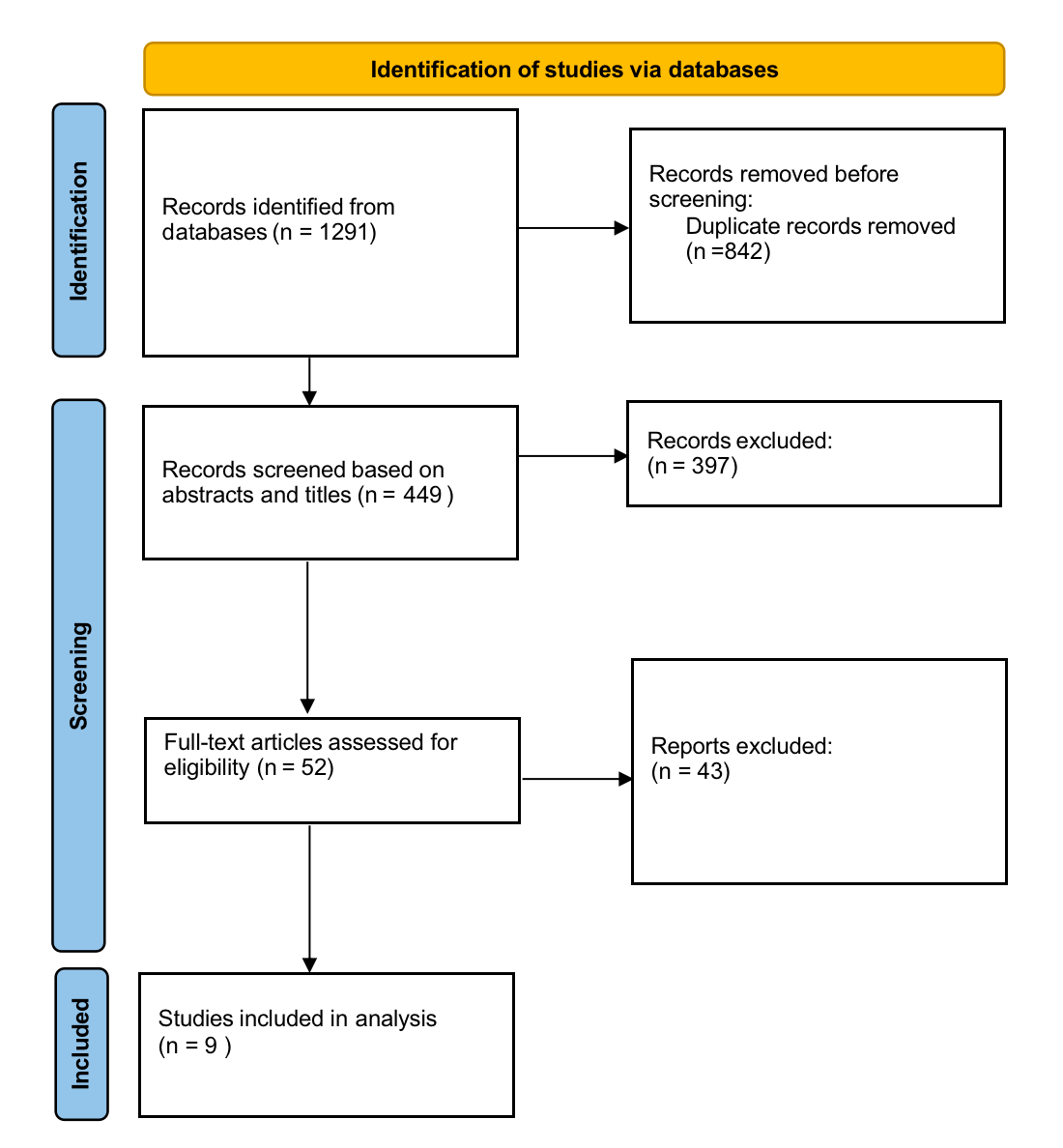


**Supplementary Figure 1. PRISMA flowchat of Literature screening**
